# Tuning the nitric oxide release from CPO-27 MOFs†
†Electronic supplementary information (ESI) available. See DOI: 10.1039/c5ra24023a



**DOI:** 10.1039/c5ra24023a

**Published:** 2016-02-03

**Authors:** Damiano Cattaneo, Stewart J. Warrender, Morven J. Duncan, Christopher J. Kelsall, Mary K. Doherty, Phillip D. Whitfield, Ian L. Megson, Russell E. Morris

**Affiliations:** a School of Chemistry, University of St Andrews, St Andrews, Fife, KY16 9ST, Scotland, UK. Email: sjw9@st-andrews.ac.uk; b Department of Diabetes & Cardiovascular Science, University of the Highlands and Islands, Centre for Health Science, Inverness, IV2 3JH, Scotland, UK

## Abstract

Nitric oxide (NO) storage and release measurements have been recorded for Ni-doped CPO-27 (Mg) and CPO-27 (Zn), and the biological effect of the released NO was assessed in porcine coronary artery relaxation tests. The results indicate that the doping strategy leads to increased levels of NO storage and delivery compared to the parent materials and that the NO dosage and biological response can be tuned *via* this approach to suit the requirements of particular applications.

## Abstract 2

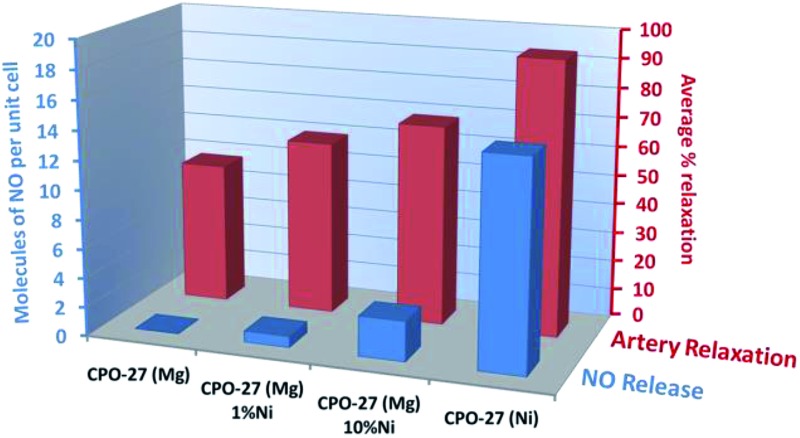
Nitric oxide release from CPO-27 MOFs and the resulting coronary artery relaxation response are tuned by isomorphous substitution of Ni into the MOF framework.

## Introduction

Nitric oxide (NO) is an important biological messenger molecule that mediates a variety of biological functions, including inhibition of platelet adhesion and aggregation, vasodilation and cell proliferation, and has also been shown to have antibacterial and wound healing properties.^1,2^ It is produced naturally in the human body *via* the conversion of the amino acid, l-arginine, to l-citrulline by the enzyme NO synthase (NOS). There are three different isoforms of NOS; eNOS (endothelial NOS) generates NO to modulate blood vessel function, nNOS (neuronal NOS) releases NO for use as a neurotransmitter in specific nerve terminals, while iNOS is an inducible enzyme that synthesises NO at high local concentrations in inflammatory cells as part of the immune defence against pathogens.^3,4^ The main parameters that determine the specific biological effect of NO are site, concentration and duration: the vasodilator, antiatherogenic and antithrombotic effects of NO are mediated by NO at very low (picomolar–nanomolar) concentrations, released by endothelial cells that line blood vessels, while the antibacterial effects are conveyed by unregulated NO synthesis from activated inflammatory cells to generate high (micromolar) local concentrations. For this reason, pharmacological products that release NO need to precisely simulate the concentration and duration of delivery at the appropriate site of the natural process associated with the condition requiring treatment. Traditional NO-delivery agents typically take the form of organic nitrates, such as glyceryl trinitrate. Other organic agents that are being developed are typically based on NONOate and *N*-nitrosothiol molecules.^5^ Such organic moieties can also be incorporated into polymer matrices to form coatings on devices.^6^ Zeolites have also been developed as storage and delivery agents for NO by binding the gas to extra-framework transition metal cations.^7^ In more recent years metal organic frameworks (MOFs) have also received much attention for this application owing to their higher storage capacities.^8^ Indeed porous materials such as zeolites and MOFs offer great potential due to their ability to release safe yet biologically active levels of the gas. They also offer the potential to help deliver the gas exactly when and where required, at the correct dosage level and for the exact length of time necessary to trigger that specific response.

MOFs can be described as nanoporous solids, formed by connecting metals with organic linking groups to form extended framework structures. These materials are well known for their high porosity and surface areas, which make them suitable for storing and delivering large quantities of gases. Additionally, their compositions, structures and functionality are also potentially tuneable, promising control over the levels of gas that can be stored and released, as well as the timescale over which this release takes place. In addition to their general porosity, particular MOF structures have been developed with features that make them even more suited to storing and releasing NO. These features include coordinatively unsaturated metal sites (CUSs) that can bind NO, or ligand moieties containing amine functionality that can form NONOate or nitroso groups.^8–12^ Among those MOFs that possess coordinatively unsaturated metal sites and that are particularly amenable to the storage and release of NO is CPO-27 (or MOF-74).^8^ CPO-27 MOFs exist as a series of isostructures that are prepared *via* the coordination of 2,5-dihydroxyterephthalic acid with different metal sources (including the three metals of interest in this study Mg, Zn, Ni^13–16^). These CPO-27 MOFs have a three dimensionally connected structure with pore sizes ∼11–12 Å. Owing to their high thermal stability and the presence of coordinated solvent molecules in the pores, these materials can be readily heated to form CUSs that are available for coordination with NO. The release of stored NO can typically be triggered by exposure to humidity whereby water exchanges for bound NO, regenerating the original hydrated material. Of the three end members considered in this study (CPO-27 (Ni), (Zn) and (Mg)) CPO-27 (Ni) has been reported to have the highest storage and delivery capacities for NO.^17^ However, due to the toxicity of nickel this material is sometimes unattractive as a candidate for certain biomedical applications. Furthermore, the level of NO that is released by CPO-27 (Ni) can even be too high for some topical applications and could lead to undesirable side effects such as inflammation. In comparison, CPO-27 (Mg) and CPO-27 (Zn) exhibit lower NO storage and release performance and show evidence of having lower toxicity, but for some applications (*e.g.* antimicrobial) the level of delivered NO is too low. Therefore in many ways the NO delivery performance and perceived toxicity of the pure end member isotypes could limit their applicability to a broad range of biomedical uses.

One of the great opportunities presented by MOFs is the potential to tune properties through metal substitution. For example, Worch *et al.* recently reported tuning the catalytic activity of a 3D coordination polymer by varying the ratio of Co/Zn in the framework.^18^ It is known that CPO-27 MOFs can be prepared in substituted forms using a variety of different metals either *via* post synthetic modification or one-step solvothermal synthesis.^19–21^ Previously reported studies have shown the benefits that can be obtained with respect to gas adsorption by doping CPO-27 MOFs with a secondary metal. For example, enhanced H_2_ adsorption was achieved from CPO-27 (Co) by doping with Ni.^20^ Interestingly, a synergistic effect was observed in which hydrogen adsorption by a sample containing 40% Ni surpassed those of the pure end members. Similar previous studies have shown that isomorphous substitution of a second metal into MOF frameworks with CPO-27 and **rtl** topologies can achieve CO_2_, H_2_ and methane adsorption values that are intermediate between those of the pure end members, suggesting that gas adsorption can be tuned by altering composition.^22,23^ Since CPO-27 (Ni) is known to store and release large quantities of NO, we postulated that doping small amounts of Ni^2+^ into the framework of CPO-27 (Mg) and (Zn) may achieve NO delivery levels intermediate between those of the pure end members, thus affording a more tuneable and controllable system that can be tailored to suit many different biomedical applications without the restrictions imposed by the properties of the pure end members. In this contribution we therefore report for the first time the use of Ni as a dopant in CPO-27 (Mg) and CPO-27 (Zn) in order to increase their NO storage and release capacities, offering a means to achieving higher NO release quantities from potentially less toxic materials. This strategy is used as a means to tune the delivered dose of NO and thereby alter the resulting biological response. This represents a significant advance in the development of MOFs as NO delivery agents for biomedical application since the achieved response will no longer be limited to that which is inducible by only the pure end members. If successful, a far wider field of use can be envisaged that encompasses the full range of NO-induced functions, whether requiring pico, nano or micro molar concentrations of NO.

## Experimental section

### Synthesis and characterisation

All reagents and solvents employed were commercially available, high-grade purity materials (Aldrich or Fluka) which were used without further purification. Pure end-member MOF samples were prepared using water-rich reflux methods based on previously reported procedures.^17,24^ In a typical procedure, Na_2_(dhtp)·2H_2_O (0.48 g, 1.72 × 10^–3^ mol) was dissolved in a mixture of deionised water and ethanol (15 mL, 1 : 1). The appropriate metal salt (Mg(NO_3_)_2_·6H_2_O, 882 mg, Zn(OAc)_2_·2H_2_O, 755 mg or Ni(OAc)_2_·4H_2_O, 856 mg) (3.44 × 10^–3^ mol), dissolved in 10 mL of water, was added to the linker solution under stirring. The mixture was refluxed for 24 hours after which the resulting products were filtered, washed with water and dried overnight in air. Ni-doped CPO-27 (Mg) and CPO-27 (Zn) were prepared through a post synthesis doping method derived from that reported by Kahr *et al.* ^19^ In this procedure Ni acetate and H_3_PO_3_ were introduced to the Mg-containing or Zn-containing mixtures described above after 24 hours under reflux (*i.e.* without isolating the pure end-member). Various Ni acetate and H_3_PO_3_ quantities were employed in order to target specific compositions in the binary Mg–Ni and Zn–Ni systems, as outlined in Table 1. The mixtures were maintained under reflux for a further 24 hours before the products were cooled, filtered, washed with water and dried overnight in air.

**Table 1:** Quantities of Ni^2+^ and H_3_PO_3_ used in the doping procedure of CPO-27 (Mg) and CPO-27 (Zn) with the resultant Ni^2+^ composition, as determined by EDX and atomic absorption analysis in the final product

Sample name	Ni^2+^ ^*a*^	H_3_PO_3_	EDX	Atomic absorption^*b*^
	(mol% on original X^*c*^)		mol% Ni/(Ni + M^*d*^)	
CPO-27 (Mg) 1% Ni	1	1	0.9	1.1
CPO-27 (Mg) 2% Ni	2	2	1.8	2.2
CPO-27 (Mg) 5% Ni	5	5	5.3	7.5
CPO-27 (Mg) 10% Ni	10	10	10.7	14.6
CPO-27 (Mg) 20% Ni	20	20	19.6	19.8
CPO-27 (Mg) 40% Ni	40	40	40.9	39.5
CPO-27 (Zn) 1% Ni	1	1	0.7	0.7
CPO-27 (Zn) 10% Ni	10	10	4.6	5.5
CPO-27 (Zn) 20% Ni	20	20	8.3	8.9

^*a*^As nickel acetate.

^*b*^All samples were digested overnight in a solution of nitric acid.

^*c*^X as Mg(NO_3_)_2_·6H_2_O or Zn(OAc)_2_·2H_2_O.

^*d*^M = Mg or Zn.

Phase purity and identity was assessed using powder X-ray diffraction (PXRD) collected on a Panalytical Empyrean diffractometer operating Cu Kα_1_ radiation monochromated with a curved Ge (111) crystal in reflectance mode. Thermogravimetric analyses (TGA) were carried out on a Perkin-Elmer Diamond *pris* Thermal Analyser at a heating rate of 5 K min^–1^ from room temperature to 873 K. Average crystal size was determined from multiple measurements of crystals in SEM images obtained on a JEOL 5600 SEM electron microscope. EDX analysis was conducted on the same SEM (both mapping and single point analysis) and compared with atomic absorption elemental analysis conducted on a Perkin-Elmer 24II elemental analyser in order to characterise the product compositions and homogeneity. NO adsorption/desorption isotherms and total release curves were collected using a bespoke gravimetric adsorption system and Sievers NOA 280i chemiluminescence nitric oxide analyser, respectively, as previously reported.^17^ Gravimetric analysis allows the quantity of NO adsorbed by each sample to be measured. During the reverse process (*i.e.* exposing the sample to vacuum) weakly bound physisorbed NO is lost from the material while any strongly bound (chemisorbed) gas remains in the sample. Any drop in mass on exposure to vacuum is used to calculate the quantity of physisorbed NO. By subtracting the physisorbed quantity from the adsorbed quantity a measure of the stored quantity of NO is determined. Release of the *stored* NO is triggered by exposing the NO-loaded MOF to a flow of humid nitrogen (11% RH). Water exchanges for NO at the metal site. By comparing the resulting total release measurements with stored quantities given by gravimetric analysis, the proportion of stored NO that can be released in this manner is calculated, giving a measure of the reversibility of CUS–NO bonding and the efficiency of the storage-release process with respect to humidity-triggered release. Collection of release data was stopped when the measured level reached 20 ppb (near the limit of the instrument resolution). TGA data were used to identify appropriate activation temperatures for each material.

### *In vitro* test

#### Vascular tissue

Pig hearts were obtained from the local abattoir, and the left anterior descending coronary arteries were dissected free of perivascular tissue and stored in physiological saline solution (PSS: NaCl 118 mM, KCl 4.7 mM, MgSO_4_ 1.2 mM, CaCl_2_ 1.25 mM, KH_2_PO_4_ 1.2 mM, NaHCO_3_ 25 mM, glucose 11 mM; pH 7.4) at 277 K for 24 hours. Segments of first order arteries of 3 mm in length were cut and mounted in a myograph (DMT model 610, Danish Myo Technologies), with or without an intact endothelium. Removal of endothelium was conducted by gentle abrasion of the artery lumen with cotton bud material.

#### Myography

Mounted arteries were maintained in PSS at 37 °C and continuously gassed with 95% O_2_/5% CO_2_ throughout the experiments. All artery segments were incubated for 45 minutes prior to normalisation by graduated stretching to achieve a resting tension of 15–20 mN. PSS containing 120 mM potassium (KPSS) was used to induce consecutive artery contractions; artery segments failing to achieve at least 60 mN force of contraction were discarded. Artery segments were subsequently pre-contracted, to around 75% of maximal contraction to KPSS, with thromboxane mimetic U46619 (1 μM) until a stable plateau was achieved, then exposed in close proximity (immediately adjacent to one end of the artery segment) to CPO-27 (Ni), CPO-27 (Mg), CPO-27 (Mg) 1% Ni or CPO-27 (Mg) 10% Ni NO-loaded MOFs for 30 minutes to allow a maximal relaxation in each case. The condition of the endothelium for each segment was tested by relaxation responses to bradykinin (10 μM).

#### Drugs

Bradykinin acetate (Sigma, UK) was dissolved at 1 mM in a 0.1 M solution of acetic acid. U46619 (Tocris, UK) was prepared in distilled water at 1 mM. Aliquots of drugs were stored at –20 °C prior to use.

#### Data analysis

Maximum relaxation responses were recorded as a % of the pre-contraction to U46619. Data are expressed as mean ± SEM. Statistical analysis was performed using 2-way ANOVA with Bonferroni *post hoc* analysis.

## Results and discussion

### CPO-27 (Mg) and Ni doped CPO-27 (Mg)

**Fig. 1:**
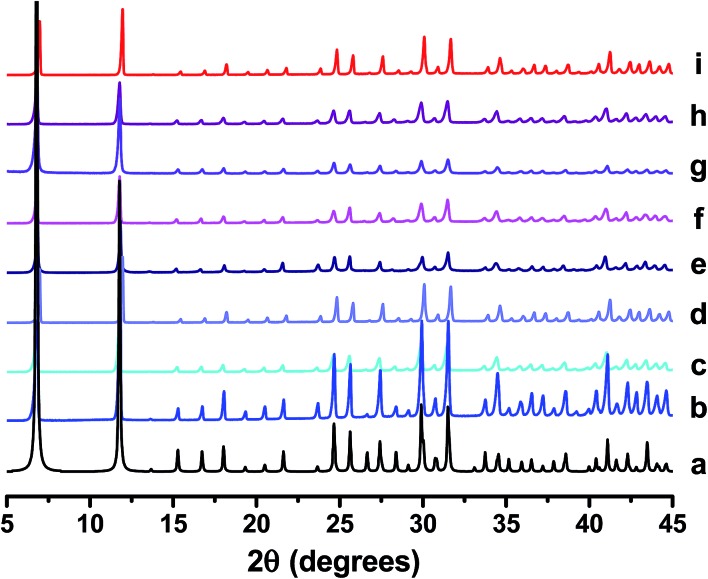
PXRD patterns of (a) reference CPO-27 (Mg),^15^ (b) CPO-27 (Mg), (c) CPO-27 (Mg) 1% Ni, (d) CPO-27 (Mg) 2% Ni, (e) CPO-27 (Mg) 5% Ni, (f) CPO-27 (Mg) 10% Ni, (g) CPO-27 (Mg) 20% Ni, (h) CPO-27 (Mg) 40% Ni and (i) CPO-27 (Ni).

CPO-27 (Mg) and CPO-27 (Ni) were prepared using standard reflux apparatus and water as the main solvent. Nickel cations were introduced into the CPO-27 (Mg) framework at different levels *via* post-synthetic treatment with an aqueous solution of Ni^2+^ acetate and a weak acid (H_3_PO_3_). For the purposes of this study it was very important to obtain doped samples with a variety of precisely targeted compositions. This two-step method was therefore chosen over recently reported one-step processes^20,21^ because such procedures were not shown to always yield product compositions matching those of the starting mixtures. This would make the targeting of specific compositions more difficult; large and wasteful excesses of metal salts would likely need to be employed to achieve targeted levels. Elemental analysis of Ni-doped CPO-27 (Mg) reported by Kahr *et al.* ^19^ suggests a more quantitative incorporation is achieved using the two-step technique. The mol% of Ni acetate and H_3_PO_3_ used in each reaction (based on molar quantity of Mg nitrate employed at the outset of the reaction) is summarised in Table 1. PXRD analysis of as-made products (Fig. 1) confirmed that each sample is phase pure. It is also confirmed that crystallinity is retained after the post-synthetic doping process.

**Fig. 2:**
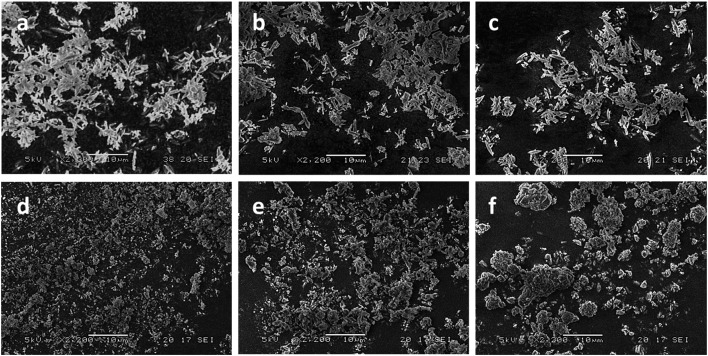
SEM images of (a) CPO-27 (Mg), (b) CPO-27 (Mg) 1% Ni, (c) CPO-27 (Mg) 5% Ni, (d) CPO-27 (Mg) 20% Ni, (e) CPO-27 (Mg) 40% Ni and (f) CPO-27 (Ni).

Consistent with our earlier observations,^17^ examination of the products by SEM revealed that pure CPO-27 (Mg) forms needle-shaped crystals with approximate average lengths of 3–7 μm (Fig. 2(a)) while pure CPO-27 (Ni) presents particles that have much smaller granulation and no discernable morphology (Fig. 2(f)). The SEM images of the Ni-doped samples show a reduction in crystal size (Fig. 2(b)–(e)) correlating with an increased Ni^2+^ loading in the synthesis. This is in contrast to observations made by Kahr *et al.* in which particle size and morphology remained relatively constant over the substituted series.^19^ This difference may be due to the higher levels of acid employed in our process, which may have resulted in greater dissolution of the original CPO-27 (Mg) end member, followed by recrystallisation of the mixed metal product. Such a process may have resulted in a more homogenous distribution of metal ions throughout the crystals, rather than the core–shell arrangement implied by Kahr (see, for example XPS results in ref. 19). Further analysis is required to probe this matter.

**Fig. 3:**
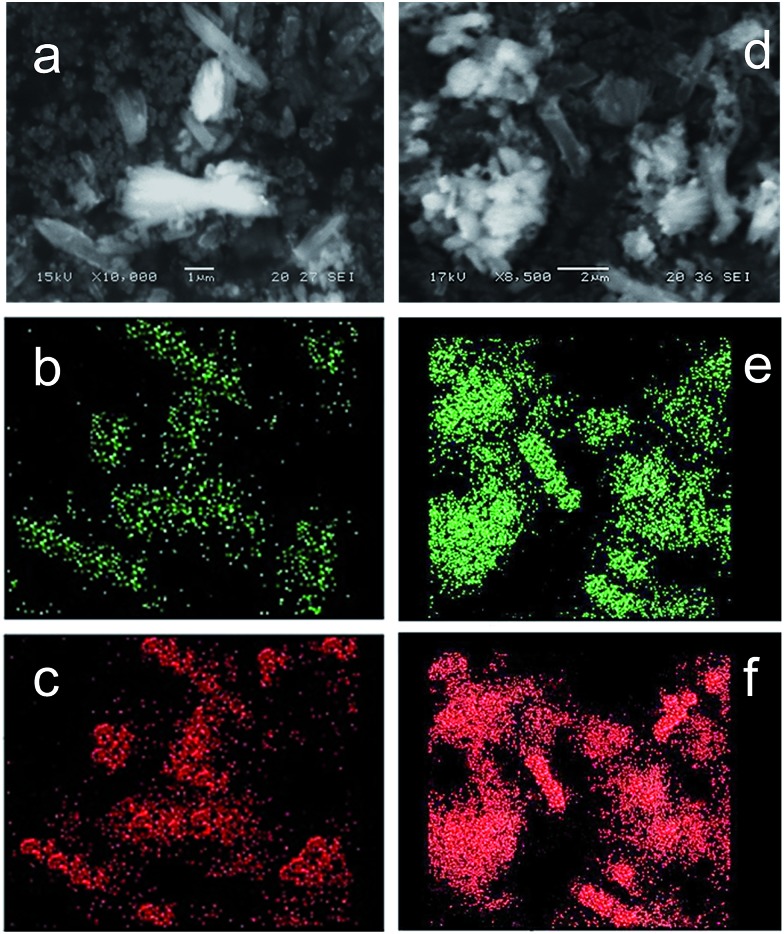
(a) SEM image of CPO-27 (Mg) 40% Ni, (b) Ni Kα elemental map and (c) Mg Kα elemental map of CPO-27 (Mg) 40% Ni, (d) SEM image of CPO-27 (Zn) 20% Ni, (e) Ni Kα elemental map and (f) Zn Kα elemental map of CPO-27 (Zn) 20% Ni.

EDX analysis of the products confirmed that Ni has indeed been incorporated into the structures at levels matching the initial concentrations used in the procedure (Table 1). The measured compositions are also consistent with the bulk composition measured by atomic absorption. This is in line with previously reported results for Ni-doped CPO-27 (Mg) prepared in this way.^19^ Results from elemental mapping of the modified frameworks (Fig. 3) show uniform and homogeneous distribution of both nickel and magnesium in the final products. The reduction in crystal size upon doping, the Ni : Mg ratios measured by EDX/AA and the elemental mapping confirm the successful incorporation of nickel in the products.

**Fig. 4:**
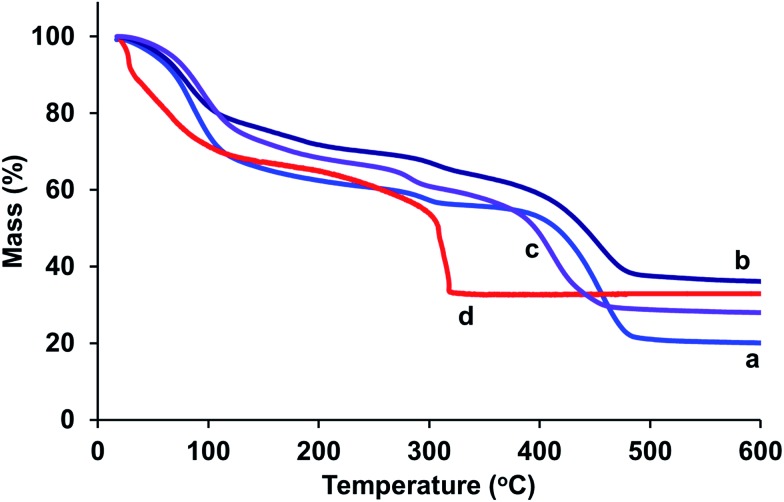
Thermogravimetric profiles of (a) CPO-27 (Mg), (b) CPO-27 (Mg) 5% Ni, (c) CPO-27 (Mg) 20% Ni and (d) CPO-27 (Ni).

Representative thermogravimetric profiles of Mg- and Ni-containing samples are shown in Fig. 4. The data indicate that Mg-rich samples have very similar thermal stability, showing loss of physisorbed water below ∼100 °C and a gradual loss of chemisorbed water up to 400 °C, after which the frameworks collapse. In contrast the thermal stability for CPO-27 (Ni) is lower, as indicated by the distinct loss in mass at ∼300 °C. The data highlight a range in temperature, between 100 and 400 °C for Mg-rich samples and between 100 and 300 °C for CPO-27 (Ni), in which the frameworks are still stable and have lost physisorbed and some (if not all) chemisorbed water (*i.e.* they are activated). The temperature of activation should be within this region and therefore since the activation is carried out under vacuum (×10^–5^ mbar), the temperature of activation was set at 150 °C.

**Fig. 5:**
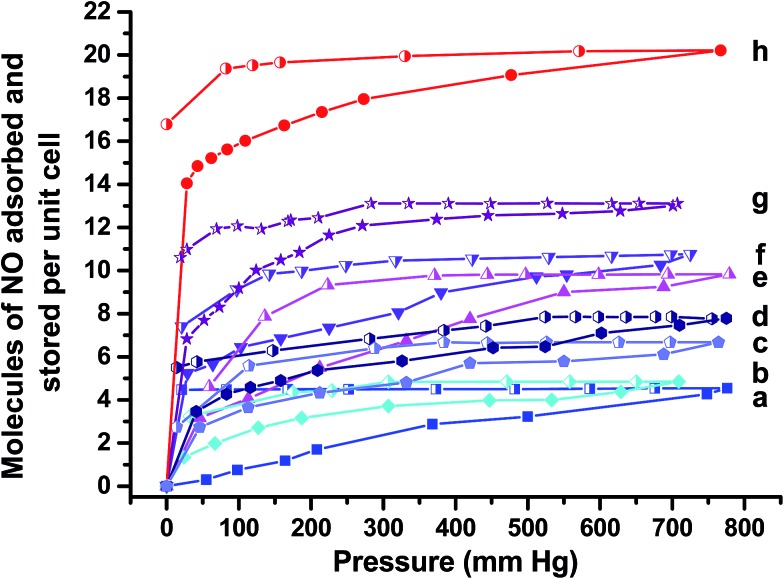
Adsorption 
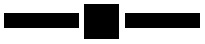
 and desorption 
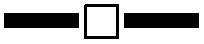
 isotherms for NO at 25 °C for (a) CPO-27 (Mg), (b) CPO-27 (Mg) 1% Ni, (c) CPO-27 (Mg) 2% Ni, (d) CPO-27 (Mg) 5% Ni, (e) CPO-27 (Mg) 10% Ni, (f) CPO-27 (Mg) 20% Ni, (g) CPO-27 (Mg) 40% Ni and (h) CPO-27 (Ni).

**Fig. 6:**
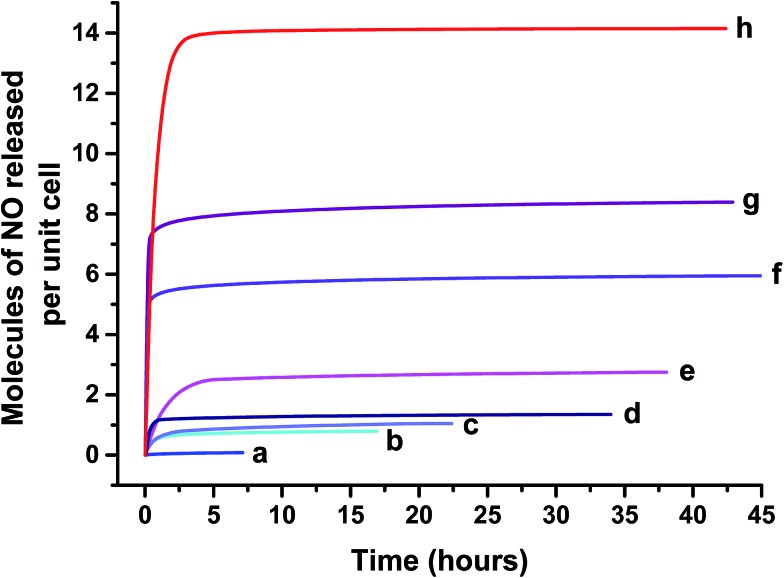
Chemiluminescence analysis of total NO delivery on contact with humid atmosphere (11% RH) for (a) CPO-27 (Mg), (b) CPO-27 (Mg) 1% Ni, (c) CPO-27 (Mg) 2% Ni, (d) CPO-27 (Mg) 5% Ni, (e) CPO-27 (Mg) 10% Ni, (f) CPO-27 (Mg) 20% Ni, (g) CPO-27 (Mg) 40% Ni and (h) CPO-27 (Ni). Data collection was stopped when NO levels reached 20 ppb.

This activation process resulted in a change of colour from yellow to pale yellow, indicating removal of physisorbed and chemisorbed water, particularly those molecules bonded to metal sites, leaving open CUSs. Exposure to NO induced a second colour change from pale yellow to dark green suggesting coordination between the metal and the radical gas.^8^ Gravimetric analysis of activated CPO-27 (Mg) confirmed that ∼4 molecules of NO per unit cell are adsorbed by this sample at room temperature (Fig. 5). The desorption isotherm of the pure magnesium framework shows a loss of 0.07 molecules of physisorbed NO per unit cell (Fig. 5) indicating near complete storage of all of the adsorbed NO. On exposure to humid nitrogen, only a very small quantity of the stored NO is released (0.1 molecules per unit cell, ∼2% of stored capacity) (Fig. 6), suggesting that the coordination of NO to Mg is irreversible when using water (at 11% RH) as a release trigger.

In contrast, and as observed in previous studies,^17^ CPO-27 (Ni) adsorbs in the region of 20 molecules per unit cell after activation (Fig. 5). The desorption isotherm indicates a loss of ∼3 molecules of physisorbed gas per unit cell and therefore a storage capacity of ∼17 molecules per unit cell. The total NO release in humid atmosphere from the pure nickel MOF is up to 14 molecules per unit cell over 40 hours (data collected to 20 ppb), highlighting an almost complete (82%) release of the stored nitric oxide. This indicates that the CUS–NO binding in this material is more labile and reversible than that in CPO-27 (Mg) with respect to water-triggered release.

The introduction of nickel to the CPO-27 (Mg) framework drastically changes the performance of the material in the adsorption, desorption, storage and release process. Incorporating up to 40% Ni into the magnesium framework increases the total quantity of NO adsorbed (from 4.5 to 13.1 molecules of NO per unit cell), NO stored (from 4.4 to 10.6 molecules of NO per unit cell) (Fig. 5) and the amount of NO released in humid conditions (from 0.1 to 8.4 molecules of NO per unit cell) (Fig. 6). Doping with Ni^2+^ also increases the time taken to reach 20 ppb levels during release experiments (the point at which release measurements are halted), up to a maximum of about 45 hours with 20% Ni. Interestingly, this timescale is not increased beyond 45 hours by doping at levels higher than 20%.

**Fig. 7:**
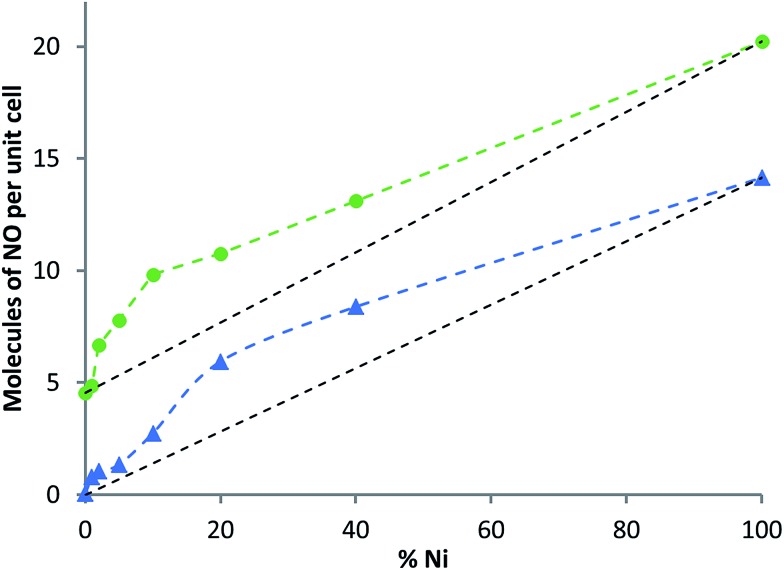
NO adsorbed (green) and total NO released (blue) *versus* increasing concentration of Ni in modified CPO-27 (Mg).

The total amount of NO adsorbed and released from the Mg-based products is plotted *versus* Ni concentration in Fig. 7. Although NO adsorption by doped samples does not surpass those of the end members (a phenomenon reported for hydrogen adsorption by Ni-doped CPO-27 (Co)^20^), the data suggest that NO adsorption and release follow non-linear relationships with the level of Ni doping, and therefore perhaps still indicate a synergistic effect. The data also indicate that the difference between the initial total adsorbed quantity of NO and that released from the quantity stored remains constant at ∼5 molecules per unit cell.

**Fig. 8:**
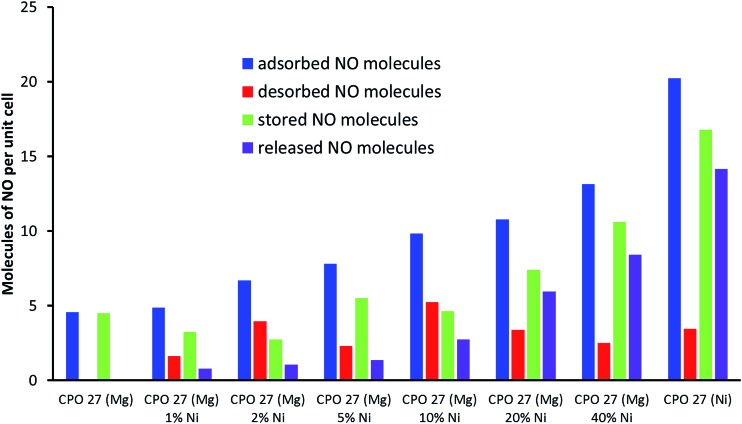
Total adsorbed (blue), desorbed (red), stored (green) (measured using a gravimetric adsorption system) and released nitric oxide (purple) at 298 K (measured using chemiluminescence analysis) for (a) CPO-27 (Mg), (b) CPO-27 (Mg) 1% Ni, (c) CPO-27 (Mg) 2% Ni, (d) CPO-27 (Mg) 5% Ni, (e) CPO-27 (Mg) 10% Ni, (f) CPO-27 (Mg) 20% Ni, (g) CPO-27 (Mg) 40% Ni and (h) CPO-27 (Ni).

**Fig. 9:**
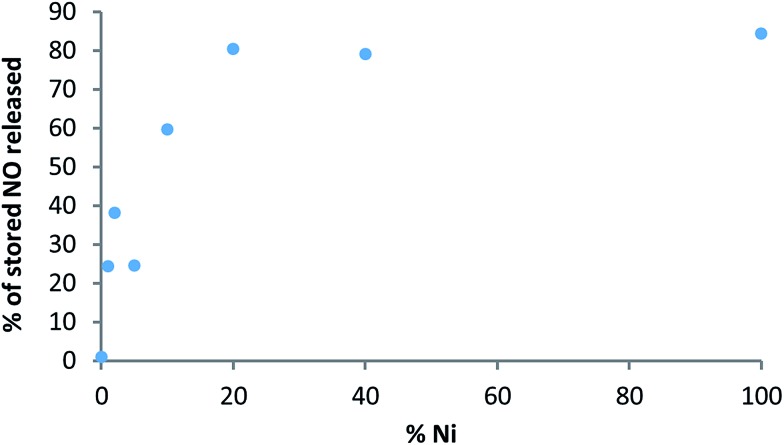
Percentage of stored NO that is released on exposure to humid nitrogen *versus* Ni content.

The variation in NO uptake, storage and release *versus* Ni doping level is further summarised in Fig. 8. The data clearly show an increase in adsorbed and released NO levels when moving to higher Ni content, as postulated at the beginning of the study. Some interesting and unexpected trends, however, are revealed when considering the changes in physisorbed and stored levels of NO. Introduction of even a very small amount of Ni (*i.e.* 1%) into CPO-27 (Mg) results in a notable increase in physisorbed NO (*i.e.* “desorbed NO” in Fig. 8, and in other words NO lost during the loading process due to the application of vacuum), and therefore a *reduction* in the quantity stored relative to pure CPO-27 (Mg). However, the proportion of the stored quantity that can be released when triggered by humidity is increased (from ∼2% to ∼25%). The increase in both physisorbed NO and the proportion of stored NO that can be released suggests that the presence of Ni in the CPO-27 (Mg) structure may make the Mg–CUS–NO bonding more labile and reversible with respect to water-triggered release. On moving to higher Ni doping levels the quantity of physisorbed NO remains reasonably constant. This, in combination with increasing adsorption levels, results in an increase in the level of *stored* NO across the series. In addition the difference between stored and released levels of NO also remains reasonably constant for all Ni-doped samples, which, in combination with the increase in stored NO, results in an increase in the proportion of stored NO that can be released as Ni content increases (Fig. 9). This may suggest that the reversibility of the CUS–NO bonding introduced by Ni^2+^ also increases with Ni content. The proportion of stored NO that is releasable, however, reaches a plateau of around 80% at and beyond Ni doping levels of 20% (Fig. 9), suggesting there is a limit to this effect. Therefore, although the released quantity still increases beyond this point (due to higher uptake and storage), there is a reduction in the delivery efficiency. Indeed, 20% Ni content in CPO-27 (Mg) appears to mark an inflexion point in NO uptake, release, release timescale and overall efficiency. Further work is underway to analyse the cause of this affect.

### CPO-27 (Zn) and Ni doped CPO-27 (Zn)

The approach described above was applied to CPO-27 (Zn) in order to assess whether it can be transferred to other related MOFs.

**Fig. 10:**
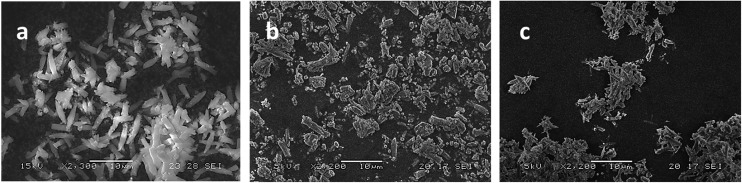
SEM images of (a) CPO-27 (Zn), (b) CPO-27 (Zn) 10% Ni and (c) CPO-27 (Zn) 20% Ni.

**Fig. 11:**
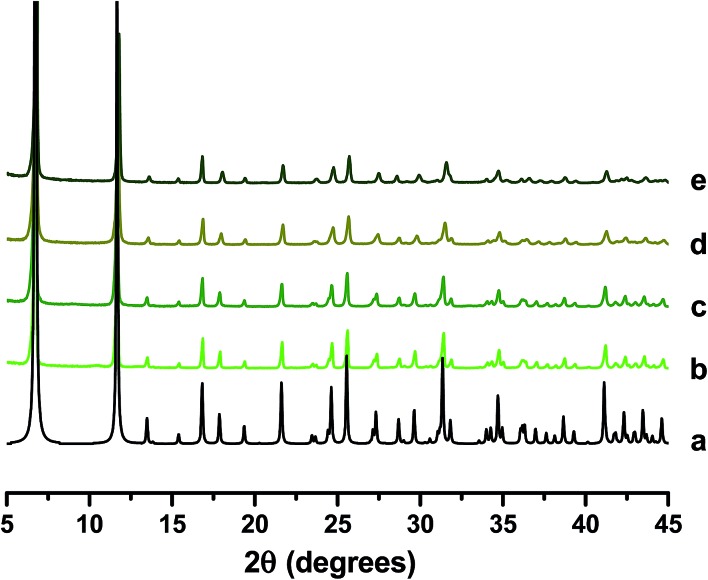
PXRD patterns of (a) reference CPO-27 (Zn),^16^ (b) CPO-27 (Zn), (c) CPO-27 (Zn) 1% Ni, (d) CPO-27 (Zn) 10% Ni and (e) CPO-27 (Zn) 20% Ni.

SEM images of the resulting products reveal some subtle differences in morphologies compared to those observed for CPO-27 (Mg). Pure CPO-27 (Zn) presents needle shaped crystals of homogenous morphology that are slightly larger than those observed for the magnesium counterpart (∼10–12 μm) (Fig. 10(a)). In contrast to CPO-27 (Mg), the modified zinc MOFs prepared *via* reflux post-treatment contain two morphologies; crystals of equal size to those of pure CPO-27 (Zn) and finer particles of less distinct morphology (Fig. 10(b) and (c)). It might be expected therefore that the two step reflux method favours the formation of a secondary phase. However, PXRD data for each product are consistent with the CPO-27 structure and show no evidence of impurities (Fig. 11).

There is also no alteration in the TGA profile (ESI Fig. 1†) on doping with Ni up to a the maximum level of doping achieved, CPO-27 (Zn) 20% Ni (measured content of 10% Ni) (Table 1). Furthermore, EDX analysis suggests that Zn and Ni are present and distributed homogenously in all crystals regardless of morphology (Fig. 3). It would therefore appear that the post synthesis reflux treatment merely results in the generation of finer particles of the same structure. We therefore hypothesise that due to the relative instability of CPO-27 (Mg) under the reaction conditions used, reflux post-treatment results in significant dissolution and re-formation leading to homogenous particle size (and size reduction concomitant with increasing Ni doping). CPO-27 (Zn), however, has slightly greater stability and does not undergo the same degree of structural collapse and re-formation. This results in greater retention of original morphology. Some dissolution does occur, however, and leads to the re-crystallisation of smaller particles.

In a further contrast to Ni-doped CPO-27 (Mg) prepared in this and previous studies using the two-step process, EDX and atomic adsorption data (Table 1) suggest that the incorporation of nickel cations in the CPO-27 (Zn) structure is less quantitative; only half of the Ni^2+^ added to the reaction mixture was included in the final product. A similar observation was made by Villajos *et al.* in Ni-doped CPO-27 (Co) prepared from a single step solvothermal reaction,^20^ and in the multi-metal CPO-27 MOFs prepared in one-step reactions by Yaghi *et al.*,^21^ suggesting that the advantage of employing the two-step doping process for CPO-27 (Mg) may not hold for all compositions. We attribute the difference in the levels of Ni doping in CPO-27 (Mg) and CPO-27 (Zn) (both prepared *via* post synthetic modification) to the higher stability of CPO-27 (Zn) under the conditions employed and its faster rate of formation compared to CPO-27 (Ni). The latter is apparent when observing one-step room temperature formation of these end member phases.^17^

**Fig. 12:**
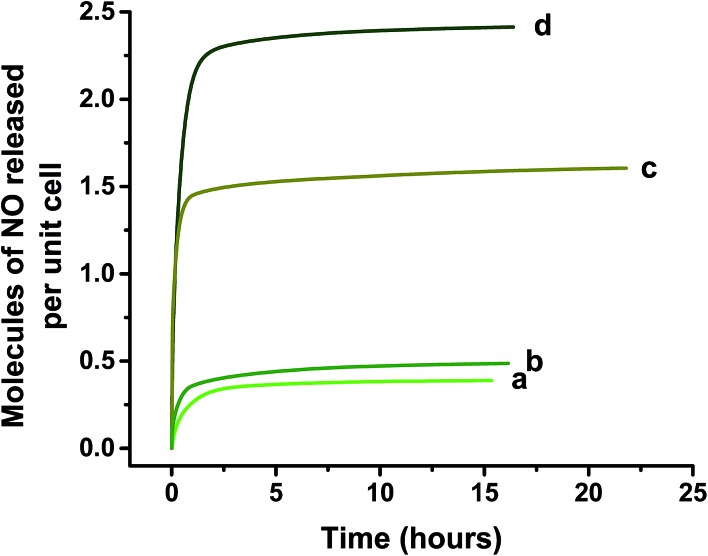
Chemiluminescence analysis of total NO delivery on contact with humid atmosphere (11% RH) for (a) CPO-27 (Zn), (b) CPO-27 (Zn) 1% Ni, (c) CPO-27 (Zn) 10% Ni and (d) CPO-27 (Zn) 20% Ni. Data collection was stopped when NO levels reached 20 ppb.

NO release properties of Ni-doped products are compared in Fig. 12. The data indicate that, as observed for CPO-27 (Mg), the introduction of Ni^2+^ into the structure markedly increases the quantity of NO released, with levels ranging from 0.3 molecules per unit cell from the unmodified MOF to 2.4 molecules per unit cell from a MOF containing a measured content of 10% Ni. Due to the absence of samples with >10% Ni (caused by the non-quantitative incorporation of Ni discussed above) it is not possible from the current data to comment on whether similar trends as those observed for CPO-27 (Mg) exist between dopant level, and adsorbed, desorbed, stored and released NO levels. Nevertheless, the data suggest that the same doping strategy employed for CPO-27 (Mg) can indeed be applied to CPO-27 (Zn) in order to enhance the NO release performance of this MOF.

### Porcine coronary artery relaxation

**Fig. 13:**
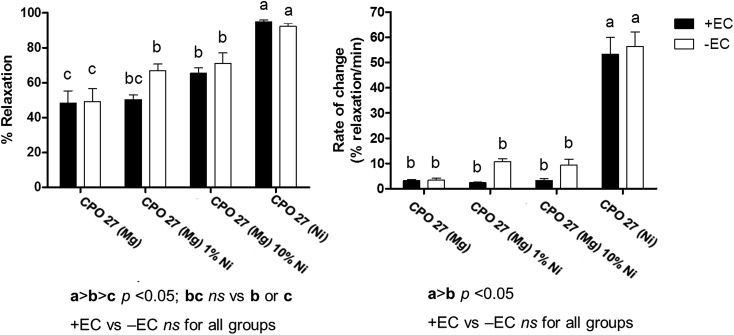
Porcine coronary artery relaxation (measured as % relaxation and rate of change) induced by NO loaded CPO-27 (Mg), CPO-27 (Mg) 1% Ni, CPO-27 (Mg) 10% Ni and CPO-27 (Ni), (Mg). +EC endothelium intact, –EC endothelium denuded, *n* = 6 per group.

Porcine coronary artery relaxation tests were conducted using four NO loaded MOF samples, namely CPO-27 (Mg), CPO-27 (Mg) 1% Ni, CPO-27 (Mg) 10% Ni and CPO-27 (Ni). These samples were selected based on NO release values measured on exposure to humid atmosphere (Fig. 8). The four samples provide a spread of release values encompassing the minimum (0.1 molecules per unit cell for CPO-27 (Mg)) and maximum (14 molecules per unit cell for CPO-27 (Ni)) levels and two low-intermediate values (0.8 molecule per unit cell for CPO-27 (Mg) 1% Ni and 2.8 molecules per unit cell for CPO-27 (Mg) 10% Ni). It is known that arterial relaxation requires only low concentrations of NO, therefore the samples were selected to give a spread of NO values weighted to the lower end of the range available to probe the sensitivity of the test system and the precision that can be achieved. The NO released from the MOFs caused varying degrees of relaxation, depending on the metal ion component; CPO-27 (Ni) caused the greatest relaxation of pig coronary arteries, followed by CPO-27 (Mg) 10% Ni, CPO-27 (Mg) 1% Ni and CPO-27 (Mg), respectively (Fig. 13 and ESI Table 1†). Samples that were not loaded with NO showed no relaxation effect (data not shown). The rate of relaxation was substantially faster in CPO-27 (Ni) treated arteries compared to all other groups; the Ni-doped CPO-27 (Mg) MOFs and CPO-27 (Mg) were comparable (Fig. 13 and ESI Table 2†). The presence or absence of an intact endothelium did not significantly affect maximal relaxation or the rate of relaxation in any group. It is notable that even pure CPO-27 (Mg) induces a relaxation response when loaded with NO despite the very low measured levels of NO released from this MOF. This highlights the very small quantities of NO required to produce biological responses.

The results indicate that biologically active levels of NO are released from these materials and that the differences in response correlate with Ni^2+^ content and the concomitant alteration of NO delivery. Most importantly, the data confirm that tailoring the composition of the MOFs in this way is a very effective means of tuning the biological response and that the response can be tuned very precisely using small alterations in MOF composition.

## Conclusion and outlook

The use of Ni^2+^ as a dopant has been shown to dramatically improve the NO release performance of CPO-27 (Mg), delivering biologically active, but non-toxic, levels of NO. In addition, the approach can be extended to CPO-27 (Zn), and we expect to other systems too. These results represent a significant advance in the development of MOFs for storage and delivery of controlled concentrations of bio-active NO because they suggest that careful manipulation of the MOF composition enables the NO concentration and resulting arterial relaxation response to be tuned; the concentration of delivered NO and the resulting biological response are no longer limited to those associated with only the pure end members. Indeed, it is envisaged that this strategy can be used to prepare materials that are able to mimic the different biological levels of NO produced in our body to target many varied specific responses, depending on the requirements of the medical situation and application. This, in turn, will broaden the spectrum of potential biomedical applications in which these materials can be utilised. This work therefore opens the door to the development and trial of MOFs as the active agent in NO-releasing medical devices, and coatings on medical devices.

Concern over the toxicity of Ni-based CPO-27 MOFs in such products should be balanced with the delivered benefit, and mitigated by judicious tuning of MOF content in the device. The results presented above offer a further means of alleviating toxicity concerns in applications requiring high NO concentrations where historically only pure CPO-27 (Ni) would be applicable; they suggest that MOFs in which Ni is partially substituted by Mg or Zn will still deliver relatively high concentrations of NO. Furthermore, many of the devices in which MOFs may be employed are only used over very short time periods, during which there is insufficient degradation of the MOF for potentially toxic leachables to be released. In other situations degradation is desirable since it allows release of antimicrobial metal ions such as Zn^2+^ and Ni^2+^. To be viable, however, a reliable and efficient method of synthesising precise compositions is required. Although the two-step doping method was adopted here due to its apparent advantage over current one-step processes, it is evident from the results for CPO-27 (Zn) that even this approach does not always yield the targeted composition. A two-step process is also more costly to run. Further work is therefore required in order to reliably obtain targeted multi-metal compositions efficiently and without having to employ large and wasteful excesses of starting reagents.
