# Supplementary material for: Tuning the nitric oxide release from CPO-27 MOFs
Source: RSC Adv. 2016 Feb 3;6(17):14059–67. doi: 10.1039/c5ra24023a (PMC4786954; doi:10.1039/c5ra24023a)
Supplement: Supplementary file 1 [file RA-006-C5RA24023A-s001.pdf]

Electronic Supplementary Material (ESI) for RSC Advances.
This journal is © The Royal Society of Chemistry 2016

Tuning the Nitric Oxide Release from CPO-27 MOFs

Damiano Cattaneo[1], Stewart J Warrender[1], Morven J Duncan[1], Christopher J Kelsall[2], Mary K Doherty[2], Phillip D Whitfield,[2] Ian L Megson[2] and Russell E Morris[1]

[1]School of Chemistry, University of St Andrews, St Andrews, Fife. KY16 9ST, Scotland.

[2] Department of Diabetes & Cardiovascular Science, University of the Highlands and Islands, Centre for Health Science, Inverness, IV2 3JH, Scotland.

Supporting Information

Figure 1: Thermogravimetric profiles of (a) CPO-27 (Zn), (b) CPO-27 (Zn) 10% Ni, (c) CPO-27 (Zn) 20% Ni and (d) CPO-27 (Ni).

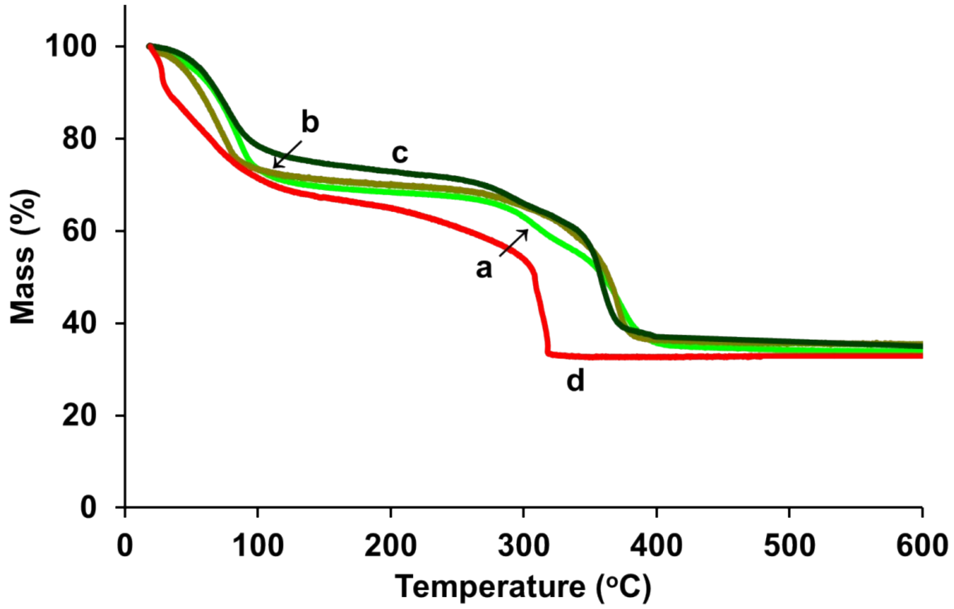

Table 1: Porcine coronary artery percentage relaxation induced by CPO-27 (Mg), CPO-27 (Ni), CPO-27 (Mg) 1% Ni and CPO-27 (Mg) 10% Ni.

**% Relaxation (maximum)**								
**CPO-27 (Ni)**		**CPO-27 (Mg)**		**CPO-27 (Mg) 1% Ni**		**CPO-27 (Mg) 10% Ni**		
**+EC**	**-EC**	**+EC**	**-EC**	**+EC**	**-EC**	**+EC**	**-EC**	
93.6	89.1	48.9	73.1	66.8	69.8	68.6	48.9	
95.1	89.1	56.1	61.5	45.2	77.1	68.4	77.7	
94.7	90.1	36,8	49.8	49.6	55.5	63.3	70.3	
92.1	96.8	21.3	25.2	41.6	61.9	74.2	83.4	
99.3	96.6	67.3	30.1	55.1	70.4	50.5	75.5	
94.9		60	54.7	51.2		67.2		
**95.0**	**92.34**	**48.4**	**49.1**	**51.6**	**66.9**	**65.4**	**71.2**	**Mean**
2.41	4.00	16.85	18.41	8.81	8.36	8.08	13.30	SD
0.98	1.79	6.88	7.51	3.60	3.74	3.30	5.95	SE

Table 2: Rate of porcine coronary artery relaxation induced by CPO-27 (Mg), CPO-27 (Ni), CPO-27 (Mg) 1% Ni and CPO-27 (Mg) 10% Ni.

**Rate of relaxation (% relaxation/min)**								
**CPO-27 (Ni)**		**CPO-27 (Mg)**		**CPO-27 (Mg) 1% Ni**		**CPO-27 (Mg) 10% Ni**		
**+EC**	**-EC**	**+EC**	**-EC**	**+EC**	**-EC**	**+EC**	**-EC**	
46.4	45.9	3.0	7.1	2.6	9.2	3.6	7.6	
40.8	74.6	4.1	3.5	2.9	14	0.7	12.8	
40.2	60	0.9	2.9	2.4	8.7	4.4	4.1	
70.2	58.8	2.5	3.1	2	13.1	5.6	5.5	
78.1	42.6	4.5	1.7	2.8	8.4	1.9	16.7	
43.8		4.0	2	1.7		3.9		
**53.23**	**56.38**	**3.15**	**3.37**	**2.40**	**10.68**	**3.35**	**9.34**	**Mean**
16.55	12.75	1.33	1.95	0.47	2.65	1.77	5.28	SD
7.40	5.70	0.54	0.80	0.19	1.19	0.72	2.36	SE
